# Deletion of Kallikrein-related peptidases ( *Klks* ) has no effect on fertility in mice

**DOI:** 10.17912/micropub.biology.001070

**Published:** 2024-01-19

**Authors:** Ryan M. Finnerty, Erin M. Carulli, Miranda L. Bernhardt, Lisette A. Maddison, Wipawee Winuthayanon

**Affiliations:** 1 OB/GYN & Women's Health, University of Missouri, Columbia, Missouri, United States; 2 Center for Reproductive Biology, Washington State University, Pullman, Washington, United States

## Abstract

Kallikreins (KLKs) are serine peptidases. It was established that *Klks* are estrogen-target genes in mouse uteri. However, the functional requirement of KLK family in the uterine function during reproduction is unknown. Here we generated a compound deletion of *Klk1b3, Klk1b4, Klk1b5,* and *Klk1* in a mouse model using CRISPR/Cas9 strategy with four single guide RNAs (sgRNAs) to target the second exon of these four genes that are aligned back-to-back in a single locus spanning 32.95 kb on chromosome 7. We found that both male and female knockout mice are fertile with no apparent health defect compared to wild-type controls. Our data suggest that *Klk1b3, Klk1b4, Klk1b5,* and *Klk1* are not necessary for male and female reproductive function in mice.

**Figure 1. Deletion of Klk1b3 , Klk1b4 , Klk1b5 , and Klk1 had no impact on fertility in male and female mice:**
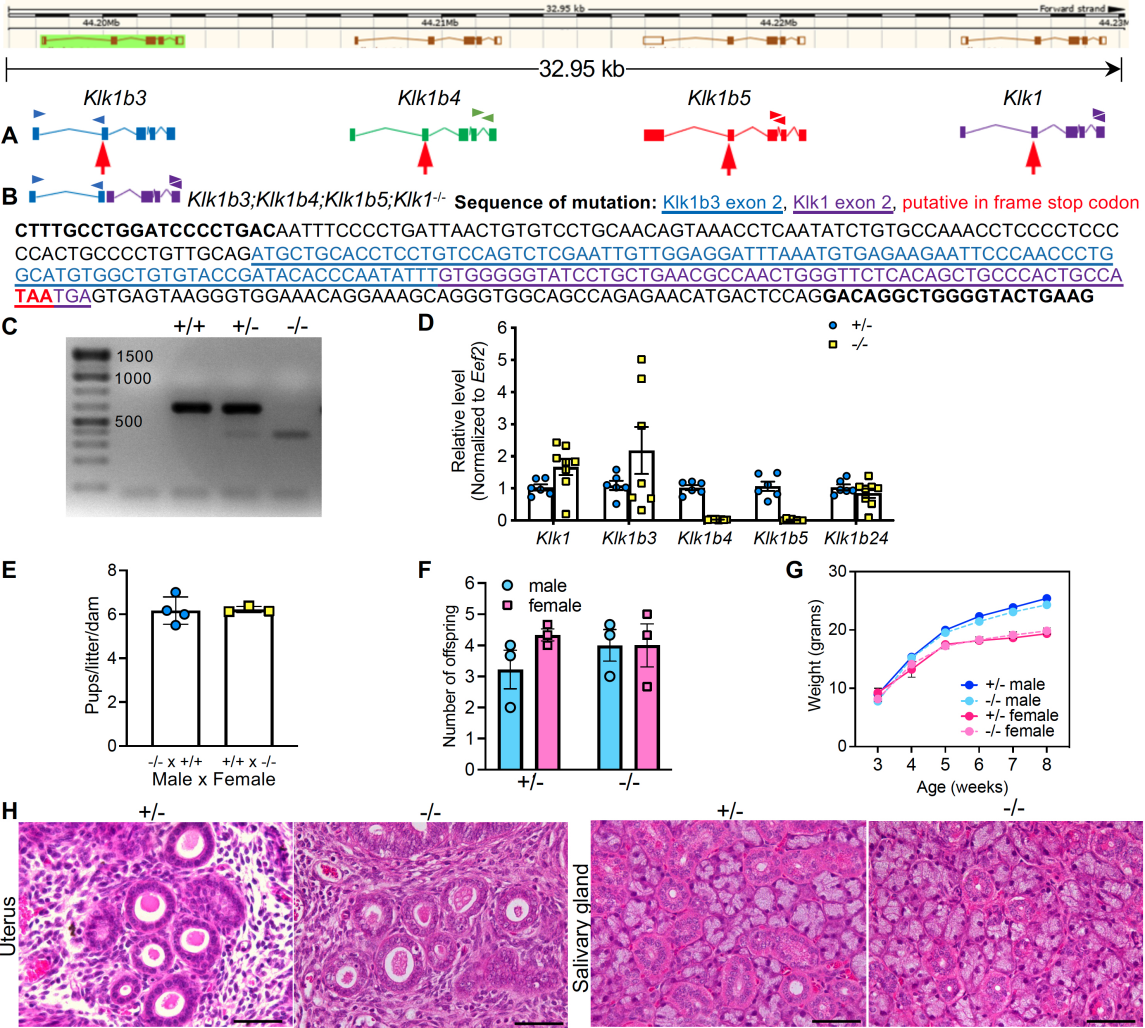
**Figure 1.** Deletion of *Klk1b3* , *Klk1b4* , *Klk1b5* , and *Klk1* had no impact on fertility in male and female mice. **A.** sgRNAs targeting exons 2 of *Klk1b3* , *Klk1b4* , *Klk1b5* , and *Klk1* (red arrows). Arrowheads above each gene indicate the locations of the forward and reverse primers used for PCR in Fig. D. **B.** Sequence of mutation in *Klk1b3* , *Klk1b4* , *Klk1b5* , *Klk1 ^-/-^* (or -/-) mice with partial *Klk1b3* and partial *Klk1* . Arrowheads the locations of *Klk1b3* and *Klk1* primers that can amplify the products in the -/- mice. **C.** Genotyping of wild-type (+/+), heterozygote (+/-), and -/- mice. **D.** Quantitative real-time reverse-transcriptase PCR of *Klk1* , *Klk1b3* , *Klk1b4* , *Klk1b5* , and *Klk1b24* (as a control) transcripts in +/- and -/- uterine samples collected at estrus. Expression levels were normalized to *Eef2* (n=6-8 mice/genotype). **E.** Fertility study of male x female (-/- x +/+ or +/+ x -/-) over 6 months. Numbers of pup/litter/dam are shown (n=3-4 mice/genotype). **F.** Number of male vs. female offspring from +/- or -/- dams (n=3 dam/genotype). **G.** Pup weight (grams) in both male and female offspring after weaning. An average weight of offspring is shown from n=3 dam/genotype. **H.** Histological analysis using H&E staining of uterine and salivary gland tissues from +/+ or -/- mice. Scale bars = 50 μm (n=3-4 mice/genotype).

## Description

Kallikreins (KLKs) are a family of trypsin- or chymotrypsin-like serine endopeptidases (Sotiropoulou et al. 2009) . These glandular KLK enzymes play roles in physiological functions such as reproduction, skin homeostasis, and neural plasticity and development (Lundwall and Brattsand 2008, Sotiropoulou et al. 2009). KLK1 is highly conserved between mammalian species (Prassas et al. 2015) . KLK2-KLK15 are also known as Kallikrein-related peptidases. Improper KLK function can lead to diseases such as hypertension, renal dysfunction, chronic inflammation, neurodegeneration, and cancer (Sotiropoulou et al. 2009) . In the uterine tissues, expression of KLK1 and KLK1-related peptidases is regulated by the sex steroid hormone, 17β-estradiol (E _2_ ) (Rajapakse et al. 2007, Iwasaki et al. 2023) . However, the role of KLKs in female reproductive function remains unclear. Our previous work showed *Klk1b5* is the most highly expressed in mouse uterus after E _2_ treatment (Li et al. 2017) . However, female mice lacking *Klk1b5* ^-/-^ are fertile and have no apparent health defect (Li et al. 2019) . In addition to *Klk1b5* , we previously reported that *Klk1b3* , *Klk1b4,* and *Klk1* are also E _2_ -target genes in mouse uteri (Winuthayanon et al. 2014) . Therefore, to functionally investigate the roles of KLK1-related peptidases (specifically *Klk1* , *Klk1b3* , *Klk1b4* and *Klk1b5* ) in reproduction, we generated a CRISPR/Cas9 modified *Klk* cluster knockout mouse model in a C57BL/6J background using four sgRNAs targeting exons 2 of *Klk1b3, Klk1b4, Klk1b5,* and *Klk1* ( **Fig. 1A** , red arrows). The cluster deletion led to a truncated locus containing partial *Klk1b3* and partial *Klk1* sequence with an in-frame stop codon, designated as *Klk1;Klk1b3;Klk1b4;Klk1b5* ^-/-^ ( **Fig. 1B** ). A 3-primer PCR approach allowed identification of knockout (-/-), heterozygous (+/-) and wild-type mice (+/+) by amplification of intact *Klk1* (larger band) or the *Klk1b3-Klk1* fusion in the knockout allele (smaller band) ( **Fig. 1C** ).

Due to the similarity across the *Klk1* gene family, the primers for *Klk1b3* amplified the sequence between exon 1 and 2, and *Klk1* primers amplified a portion of exon 5 to obtain specificity. These portions of the genes remained in the -/- animals, however, and the corresponding amplicons for *Klk1b3* and *Klk1* amplicons were detected in -/- uteri ( **Fig. 1D** ). This was not an indication that the genes remained intact or that a protein made. The *Klk1b4* and *Klk1b5* transcripts were not detected in -/- samples. *Klk1b24* , another E _2_ -target gene, was used as a control, and we found that the level of *Klk1b24* in the -/- mouse uterus was not increased as a compensatory mechanism. However, we did not evaluate the expression of the *Klk1* family in other cell types. Therefore, it is unclear whether there are compensatory mechanisms in other cell types in -/- mice. It is unlikely the *Klk1b3-Klk1* fusion would result in a functional protein. Due to the premature stop codon shortly after the fusion, there would be 49 amino acids of KLK1B3, and 17 novel amino acids since the KLK1 amino acid sequence is out of frame. In the unlikely case that protein is made, it would be missing the peptidase domain, rendering an inactive enzyme function. As described below, we have not observed any phenotype nor an abnormality, we reason that even if the fusion protein was made, the novel protein does not have biological function *in vivo* .

A 6-month fertility trial showed that both -/- males and females are fertile with an average of 6 pups per litter ( **Fig. 1E** ). There was no significant difference between male vs. female progeny from +/- or -/- dams ( **Fig. 1F** ). Both male and female +/- and -/- pups showed similar weight gain from 3 weeks old to adulthood (8-week-old) ( **Fig. 1G** ). Uterine gland and salivary gland were evaluated in adult female mice collected at the estrus stage of ovarian cycle. There were no apparent histological differences between +/- or -/- tissues ( **Fig. 1H** ). Our investigation has revealed that compound deletions of *Klk1;Klk1b3;Klk1b4;Klk1b5* have no effect on fertility in male or female mice. It suggests that the function of genes in a large family such as *Klk1* is difficult to evaluate due to its functional redundancy.

## Methods

Capped Cas9 mRNA was purchased from TriLink BioTechnologies and sgRNAs were generated using a cloning-free method and in-vitro transcription (ThermoFisher). Hematoxylin and Eosin were purchased from Fisher Scientific. sgRNA sequences for *Klk1b3* , *Klk1b4* , *Klk1b5* , and *Klk1* are as followed: CGATACACCCAATATTTATG CGG, CAGTCCACTTGAGACTGGAC AGG, GAGTCCTGCTGAACGCCAAC TGG, and CTTCACCAAATATCAATGTG GGG. C57BL/6J mice were purchased from Jackson Laboratory. A mixture of all 4 sgRNAs and Cas9 mRNA was injected into the pronucleus of zygotes and the injected embryos were surgically transferred to the oviducts of pseudopregnant females. Genotyping primers include MutationF CTGTGTACCGATACACCCAATATTGG, Klk1F CCTGTCATCCCTTAGCCCAA, and Klk1R ACACATGAGACCCAGAACCA; 693bp WT-allele (Klk1F-R) and 367bp deletion allele (MutationF-Klk1R). AccuStart II PCR SuperMix (Quanta Bioscience) was used for genotyping. PCR reactions are as followed: 1) 94°C 3:00 min, 2) 30 cycles of 94°C 0:30 sec, 55°C 0:30 sec, 72°C 0:45 sec, 3) 72°C 5:00 min, 4) 16°C 5:00 min, and 5) 4°C hold. Quantitative real-time reverse-transcriptase PCR was performed using the method described previously (Li et al. 2019) .
